# ESBL Producing *Escherichia coli* in Faecal Sludge Treatment Plants: An Invisible Threat to Public Health in Rohingya Camps, Cox's Bazar, Bangladesh

**DOI:** 10.3389/fpubh.2021.783019

**Published:** 2021-12-15

**Authors:** Md. Sakib Hossain, Sobur Ali, Monir Hossain, Salman Zahir Uddin, M. Moniruzzaman, Mohammad Rafiqul Islam, Abdullah Mohammad Shohael, Md. Shafiqul Islam, Tazrina Habib Ananya, Md. Mominur Rahman, Mohammad Ashfaqur Rahman, Martin Worth, Dinesh Mondal, Zahid Hayat Mahmud

**Affiliations:** ^1^International Centre for Diarrhoeal Disease Research, Dhaka, Bangladesh; ^2^Department of Biotechnology and Genetic Engineering, Jahangirnagar University, Dhaka, Bangladesh; ^3^WASH Section, United Nations Children's Fund, Dhaka, Bangladesh; ^4^Department of Chemical Engineering, Bangladesh University of Engineering and Technology (BUET), Dhaka, Bangladesh

**Keywords:** ESBL-producing *E. coli*, multidrug-resistant, Rohingya camps, Bangladesh, faecal sludge

## Abstract

**Introduction:** Human faecal sludge contains diverse harmful microorganisms, making it hazardous to the environment and public health if it is discharged untreated. Faecal sludge is one of the major sources of *E. coli* that can produce extended-spectrum β-lactamases (ESBLs).

**Objective:** This study aimed to investigate the prevalence and molecular characterization of ESBL-producing *E. coli* in faecal sludge samples collected from faecal sludge treatment plants (FSTPs) in Rohingya camps, Bangladesh.

**Methods:** ESBL producing *E. coli* were screened by cultural as well as molecular methods and further characterized for their major ESBL genes, plasmid profiles, pathotypes, antibiotic resistance patterns, conjugation ability, and genetic similarity.

**Results:** Of 296 isolates, 180 were phenotypically positive for ESBL. All the isolates, except one, contained at least one ESBL gene that was tested (*bla*_*CTX*−*M*−1_, *bla*_*CTX*−*M*−2_, *bla*_*CTX*−*M*−8_, *bla*_*CTX*−*M*−9_, *bla*_*CTX*−*M*−15_, *bla*_*CTX*−*M*−25_, *bla*_*TEM*_, and *bla*_*SHV*_). From plasmid profiling, it was observed that plasmids of 1–211 MDa were found in 84% (151/180) of the isolates. Besides, 13% (24/180) of the isolates possessed diarrhoeagenic virulence genes. From the remaining isolates, around 51% (79/156) harbored at least one virulence gene that is associated with the extraintestinal pathogenicity of *E. coli*. Moreover, 4% (3/156) of the isolates were detected to be potential extraintestinal pathogenic *E. coli* (ExPEC) strains. Additionally, all the diarrhoeagenic and ExPEC strains showed resistance to three or more antibiotic groups which indicate their multidrug-resistant potential. ERIC-PCR differentiated these pathogenic isolates into seven clusters. In addition to this, 16 out of 35 tested isolates transferred plasmids of 32–112 MDa to *E. coli* J53 recipient strain.

**Conclusion:** The present study implies that the faecal sludge samples examined here could be a potential origin for spreading MDR pathogenic ESBL-producing *E. coli*. The exposure of Rohingya individuals, living in overcrowded camps, to these organisms poses a severe threat to their health.

## Introduction

Antibiotic resistance is one of the most concerning present global issues and it is increasing at an alarming rate. Nowadays, it is one of the major threats to global public health and WHO enlisted it as one of the top ten threats in 2019 (1). Excessive use or misuse of broad-range antibiotics in medicine and agriculture is the most prominent reason for the development of antibiotic resistance. The incomplete metabolism in human bodies leads to the release of a significant amount of antibiotics into faecal sludge and subsequently in the environment (2). Moreover, in faecal sludge treatment plants (FSTPs), chemical, and biological treatment processes have the potential to promote the development of antibiotic resistance and their subsequent dissemination. Bacteria can attain antimicrobial resistance (AMR) through many mechanisms, including enzymatic inactivation of antibiotics, target modification, and dynamic efflux. Enzymatic cleavage utilizing β-lactamses specially by ESBLs is a powerful mechanism of acquired resistance against broad-range of β-lactam antimicrobials (3). ESBLs can hydrolyze β-lactam antibiotics including first, second, third and even fourth-generation cephalosporins, and aztreonam which are susceptible to β-lactamase inhibitors (4). ESBL production is one of the most familiar procedures to gain resistance against β-lactam antibiotics of Enterobacteriaceae including *E. coli* and *Klebsiella pneumoniae* (5, 6). Predominantly, there are three types of ESBL enzymes, such as CTX-M, SHV, and TEM, which exhibit 25% homology among them (5, 7). The genes of these various ESBLs are mostly plasmid-coded and can disseminate through horizontal gene transfer between bacteria and even between different species (8). With this process, pathogens can acquire resistance genes from environmental bacteria (9–12).

Commensal *E. coli* is a common inhabitant of the intestines of humans and different animals, and some *E. coli* strains are pathogenic to both humans and animals and can even lead to the onset of life-threatening infections (3). To date, six well-described diarrhoeagenic *E. coli* pathotypes have been identified that can infect humans (13): enteropathogenic *E. coli* (EPEC), enteroinvasive *E. coli* (EIEC), enterohemorrhagic *E. coli* (EHEC), enteroaggregative *E. coli* (EAEC), enterotoxigenic *E. coli* (ETEC), and diffusely adherent *E. coli* (DAEC). Besides, urinary tract infection (UTI) is one of the most common extraintestinal *E. coli* infections and the causative agent is uropathogenic *E. coli* (UPEC). The pathotype, meningitis-associated *E. coli* (MNEC), can cause meningitis and sepsis, another reason for extraintestinal infections that are becoming more common. The *E. coli* pathotypes with the ability to cause extraintestinal diseases are known as ExPECs (14).

In developing countries, antibiotic resistance to enteric pathogens is of particular concern as it is considered one of the most significant challenges to treat infectious diseases. Several studies have reported the isolation of pathogenic as well as resistant *E. coli* from environmental samples, which can participate in horizontal gene transfer of their resistance genes containing plasmids and lead to outbreaks in a densely populated area (15–20) like Rohingya camps. Over 330 FSTPs of different technologies have been established within the camps to treat the faecal sludge properly before discharging into the environment. These technologies treat the sewage emptied from the thousands of pit latrines in the Rohingya camps. Personal and domestic hygiene are not adequate there, which poses a serious threat of introducing pathogens directly into the environment and thus creating the possibilities for outbreaks.

Faecal sludge is one of the most extensive repositories of antibiotic resistance because of the introduction of antibiotic-resistant bacteria (ARB) and antibiotic resistance genes (ARGs) from human faecal materials. Horizontal gene transfer may be facilitated by the high concentration and diversity of microbial flora of FSTPs *via* mobile genetic components like plasmids (21, 22). It is alarming that, in many studies, the existence of an increasing ratio of ARB was reported both in raw sludge and effluent of the treatment plants in the case of urban sewage (23–25) as well as hospital effluents (26, 27). However, apart from medicinal residues and ARGs, the main focus of faecal sludge recycling was on heavy metals, bacterial pathogens, and organic contaminants. And it is a matter of great concern that treatment processes do not always remove infectious pathogens efficiently, which may make their entry into the soil and food chain again along with their antibiotic-resistance properties through treated wastewater and faecal sludge if used for agricultural purposes (28–30). ARGs are also found in biosolids-enriched soils and decay much slowly (31–33). The aim of this study was to describe the occurrence of ESBL-producing *E. coli* before and after treatment in different FSTPs in the Rohingya camps. The investigation was also intended to determine the pathogenic genes in ESBL producing *E. coli* and their antibiotic resistance pattern. A conjugation experiment was also conducted to see the gene transfer capability of the ESBL-*E. coli* and ERIC-PCR was carried out to explore the genetic homogeneity among the pathogenic ESBL-*E. coli* isolates.

## Materials and Methods

### Sampling Sites and Sample Collection

**Figure 1:**
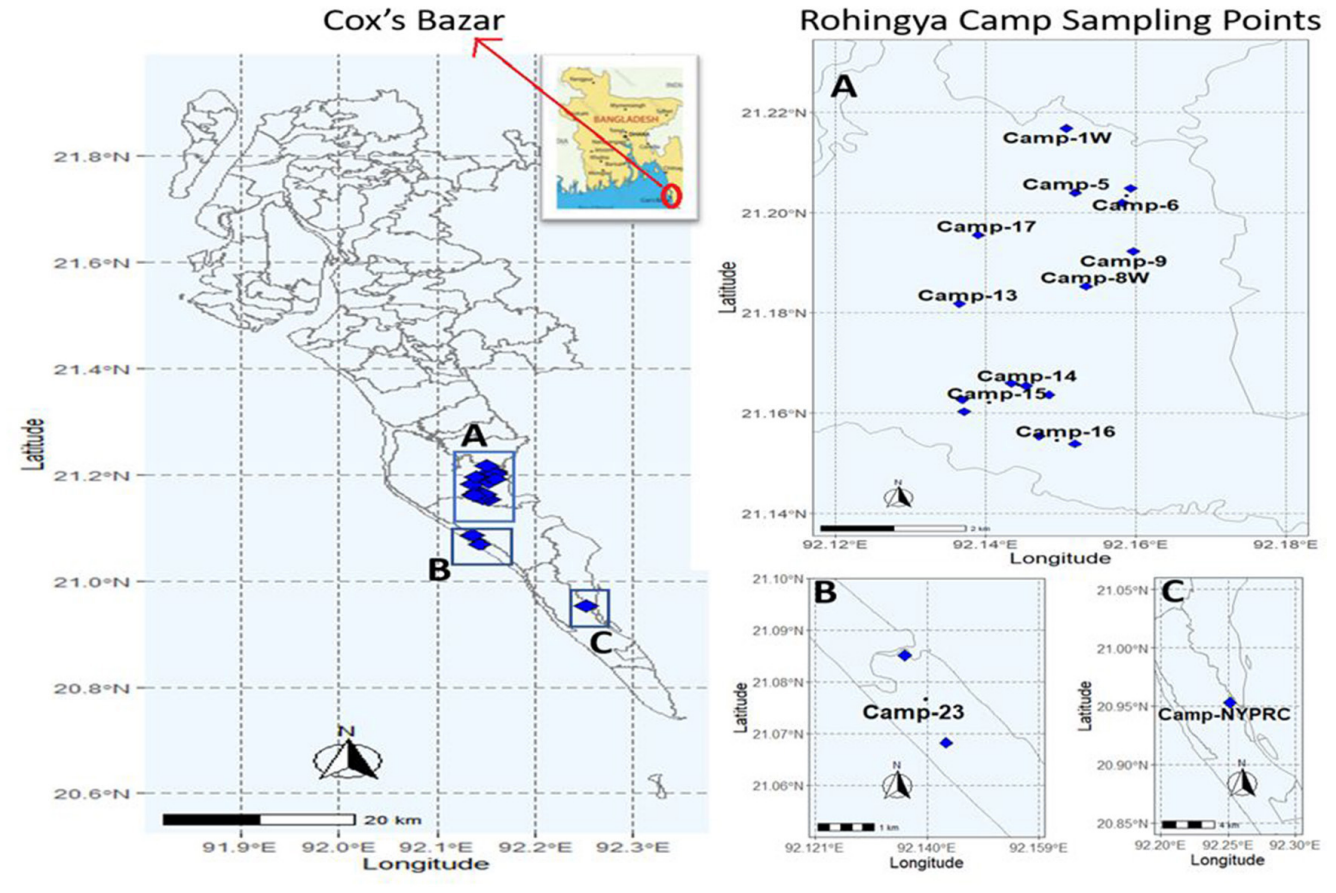
Locations of sampling points in Rohingya camps, Cox's Bazar.

This study was to investigate the FSTPs at the Rohingya camps, Cox's Bazar (Figure 1). Sampling was done with proper approval from the Refugee Relief and Repatriation Commissioner (RRRC). Samples were collected from eighteen different FSTPs of six different technologies (i.e., upflow filter, constructed wetland, anaerobic baffled reactor, lime stabilization pond, wastewater stabilization pond, and decentralized wastewater treatment system) in the period between November 2019 to January 2020. Two sludge samples- inlet (before treatment) and outlet (after treatment) were collected from each FSTP. Altogether, 108 FSTP samples were collected from both inlets and outlets of the plants in 3 consecutive rounds consisting of 36 samples in each round. Approximately, 500 mL sample was collected in a sterile 500 mL plastic bottle (NALGENE, NY, USA) with proper label. After collection, the samples were shipped by air transportation from Cox's Bazar to the Laboratory of Environmental Health, icddr,b, Dhaka in an insulated box maintaining a temperature ranging from 4 to 10°C (33, 34).

### Sample Processing

Samples were processed within 24 h after collection, maintaining standard procedure. For analysis, faecal sludge samples were subjected to serial dilutions using autoclaved normal saline (0.85% NaCl). Hundred milliliter of serial decimal dilutions (1/10, 1/100, 1/1,000, 1/10,000, 1/100,000, 1/1,000,000) of the samples were filtered through a 0.22 μm membrane filter (Sartorius Stedim, Goettingen, Germany) in a Millipore filter unit (Millipore, Darmstadt, Germany). The membrane filters were then firmly placed on a modified Thermotolerant *E. coli* (mTEC) agar (BD Difco, NJ, USA) plate. Subsequently, at 35 ± 0.5°C, the culture plate was incubated for 2 h, followed by another episode of incubation at 44.5 ± 0.2°C for 22 ± 2 h.

### Isolation and Identification of ESBL and KPC Positive *E. coli*

After incubation, colonies with red to magenta color on mTEC media were considered as presumptive *E. coli*. Further, 296 isolated colonies were picked and inoculated on CHROMagar ESBL^TM^ (CHROMagar, Paris, France) and CHROMagar KPC^TM^ (CHROMagar, Paris, France) media by patch inoculation method and incubated at 37°C for 18–24 h. Growth and distinctive colony color on these media confirm extended-spectrum β-lactamases (ESBLs) and *Klebsiella pneumoniae* carbapenemase (KPC) production by the isolates, respectively. Dark pink to reddish colonies on both CHROMagar ESBL^TM^ and CHROMagar KPC^TM^ represent *E. coli* that can produce ESBLs and Carbapenemases, respectively (34). Further, isolates were confirmed by using the *API 20E* kit (Biomerieux SA, Marcy-I'Etoile, France) as per the manufacturer's protocol. After confirmation, a stock culture supplemented with 30% (v/v) glycerol was prepared from an enrichment culture of respective isolate and stored at −80°C for further investigation. One loopful from the stock culture was taken, streaked on MacConkey agar (BD Difco, NJ, USA) and incubated for 16–18 h at 37°C for bacterial revival.

### Bacterial Cell Lysate Preparation

DNA from the samples was prepared following the boiling lysis method (35). For this purpose, one or two discrete colonies were taken from the MacConkey agar plate of pure culture and inoculated into 3 ml of LB broth, incubated overnight at 37°C, 120 rpm in the innova™ 4300 incubator shaker (NEW BRUNSWICK SCIENTIFIC, NJ, USA). Then, 1.5 ml of fresh culture was taken and centrifuged at 13,000 rpm for 5 min. The supernatant was discarded, and the pellet was resuspended into 600 μL of autoclaved distilled water and mixed well by pipetting. Immediately, after boiling at 100°C for 10 min on Stuart^®^ block heater (Cole-Parmer, Stone, UK), it was then cooled in ice for 10 min and centrifuged at 13,000 rpm for 7–8 min. Finally, 100 μL from the supernatant was stored at −20°C.

### Detection of the *bla* Genes by PCR

The total DNA content of the isolates was used to test the presence of *bla* gene groups, *bla*_*CTX*−*M*−1_, *bla*_*CTX*−*M*−2_ (36), *bla*_*CTX*−*M*−8_(37), *bla*_*CTX*−*M*−9_ (36), *bla*_*CTX*−*M*−15_ (38), *bla*_*CTX*−*M*−25_ (39), *bla*_*TEM*_ (40), and *bla*_*SHV*_ (41) in all the isolates by polymerase chain reaction as per methods described before. All the PCR reactions were conducted on Bio-Rad T100™ Thermal Cycler (Bio-Rad, CA, USA). For individual reactions, separate primer sets were used. The primer sequences and their corresponding PCR products specifying different antibiotic resistance genes are listed in Supplementary Table 1. Agarose gel electrophoresis was performed for the separation of PCR products with 1% agarose gel.

### Plasmid Isolation and Profiling

Plasmids were extracted from the ESBL producing *E. coli* isolates following the Kado alkaline lysis procedure with slight modifications (42). In brief, the extracted plasmids were analyzed with a horizontal electrophoresis technique employing 0.7% agarose gel and 1X Tris Borate EDTA (TBE) buffer. Molecular sizes were determined by comparing the differential distance of bands in the gel of the unknown sample with the size standard plasmids. The plasmids that were used as size standards were Sa (23 MDa), RP4 (36 MDa), R1 (62 MDa), PDK9 (2.1, 2.7, 105, 140 MDa), and *E. coli* V517 (1.4, 1.8, 2.0, 2.6, 3.4, 3.7, 4.8, 35.8 MDa) (43). Plasmids were mixed with 6X loading dye before stacking into the gel and the voltage was set to 100 V to carry out the electrophoresis.

### Detection of Diarrhoeagenic *E. coli*

All confirmed ESBL producing *E. coli* isolates were further analyzed for the presence of diarrhoeagenic genes by PCR. Certain genes encoding virulence factors were selected for the detection of diarrhoeagenic *E. coli* e.g., anti-aggregation protein transporter (*aat*) and aggR-activated island (*aaiC*) for EAEC, attaching and effacing (*eae*) and bundle forming pilus (*bfp*) for EPEC, heat-labile (*lt*) and heat-stable (*st*) for ETEC (44). PCR was also performed for the detection of invasion plasmid antigen H (*ipaH*) and the invasion-associated locus (*ial*) for EIEC (45, 46). The multiplex PCR reaction was carried out following the protocol described before (35). The second multiplex PCR to detect genes encoding Shiga toxin (*stx1* and *stx2*) was conducted according to the previously published procedure (47, 48). The sequences of primers and corresponding product lengths are listed in Supplementary Table 1.

### Detection of Extraintestinal Pathogenic *E. coli* (ExPEC)

The non-diarrhoeagenic isolates were subsequently tested for seven extraintestinal pathogenic *E. coli* (ExPEC) related virulence genes. The pathogenic markers are: *afa* (afimbrial adhesins), *focG* (F1C fimbriae), *hlyD* (cytolytic protein toxin), *iutA* (iron acquisition system), *kpsMII* (group 2 polysaccharide capsule), *papA* (P fimbriae), and *sfaS* (S fimbriae) (49, 50). Two multiplex PCR were performed for the detection of these genes (51). If three or more of these genes are present in one isolate, that can be characterized as an ExPEC strain (52).

### Determination of Antibiotic Susceptibility Patterns

The patterns of antibiotic susceptibility for the pathogenic, both diarrhoeagenic and ExPEC gene harboring isolates, were obtained by following the standard Kirby-Bauer disk diffusion method as per the suggestion of the Clinical and Laboratory Standards Institute (CLSI) guidelines (53). Interpretation of antimicrobial susceptibility patterns was made for 19 antibiotic agents. For antimicrobial susceptibility testing, commercially available antibiotic disks (Thermo Scientific™ Oxoid™, Basingstoke, Hampshire, UK) e.g., ampicillin (AMP, 10 μg), cefuroxime (CXM, 30 μg), ceftazidime (CAZ, 30 μg), cefotaxime (CTX, 30 μg), cefepime (FEP, 30 μg), aztreonam (ATM, 30 μg), imipenem (IPM, 10 μg), meropenem (MEM, 10 μg), amikacin (AK, 30 μg), gentamicin (CN, 10 μg), nitrofurantoin (F, 300 μg), tetracycline (TE, 30 μg), tigecycline (TG, 15 μg), nalidixic acid (NA, 30 μg), ciprofloxacin (CIP, 5 μg), sulfamethoxazole-trimethoprim (SXT, 25 μg), chloramphenicol (C, 30 μg), azithromycin (AZM, 15 μg), and fosfomycin (FOS, 50 μg) were used. In this experiment, we used *E. coli* ATCC 25922 strain as a standard organism, and the procedure was replicated three times. The average measurement of the diameter of inhibition zone (mm) for individual antibiotic agent was determined and from this information, isolates were categorized as susceptible, intermediate, and resistant as per CLSI guidelines (53).

### DNA Fingerprinting by ERIC-PCR

For the investigation of genetic relatedness among the pathogenic isolates, Enterobacterial Repetitive Intergenic Consensus (ERIC) sequences PCR was performed using ERIC2 primer (5′-AAGTAAGTGACTGGGGTGAGCG-3′). The PCR was carried out as per the protocol mentioned before (54). The amplified PCR products were resolved on 2% agarose gel. Gels were run at 90 volts for ~3 h. Invitrogen 1 kb plus ladder (Thermo Fisher Scientific, US) was used in the very first and last lanes per gel. GelJ v.2.0 software was used for gel image analysis (55). The gaussian regression method was used to normalize the image. Clusters of ERIC-PCR patterns were generated through dice coefficient and the unweighted pair group method using arithmetic averages (UPGMA) with 1.0% tolerance value.

### Conjugation Ability Testing

In the conjugation experiment, donor isolates were selected based on their plasmid profiles from the pathogenic and non-pathogenic isolates. The non-pathogenic isolates were picked randomly from different sampling rounds. Usually, a threshold size of 50 MDa was selected as a selection criterion as large plasmids containing antimicrobial-resistant genes are responsible for drug resistance (56). Twenty-two pathogenic and thirteen non-pathogenic isolates were taken as donors. Sodium azide-resistant *E. coli*-J53 was used as the recipient. Conjugation was performed in broth mating assay at 30°C for 19 ±1 h. MacConkey agar plates containing cefotaxime (1 mg/L) and sodium azide (100 mg/L) were used to select transconjugants based on growth and colony morphology. Transconjugants were verified by plasmid profiling, antibiotic susceptibility tests by disk diffusion method and the presence of ESBL genes by PCR.

## Results

### Frequent Occurrence of ESBL-*E. coli* in Faecal Sludge Samples

A total of 108 isolates from inlets and 188 isolates from outlets were inoculated in CHROMagar ESBL^TM^ and CHROMagar KPC^TM^ media by patch inoculation method for the phenotypic identification of ESBL and KPC positive *E. coli*. Finally, 180 ESBL producing *E. coli* isolates were identified on CHROMagar ESBL^TM^ agar plates, where 69% (75/108) of the isolates were from inlets and 56% (105/188) from the outlets. The overall distribution of ESBL producing *E. coli* was 61% (180/296). These isolates were recovered from 78% (42/54) of the inlets and 69% (37/54) of the outlet samples. Interestingly, none of the isolates was found positive for KPC. The counts of *E. coli* isolates were represented in Supplementary Table 2.

### *bla_*CTX*−*M*−1_* and *bla_*CTX*−*M*−15_* Genes Were More Prominent in ESBL-*E. coli* Isolates

**Figure 2:**
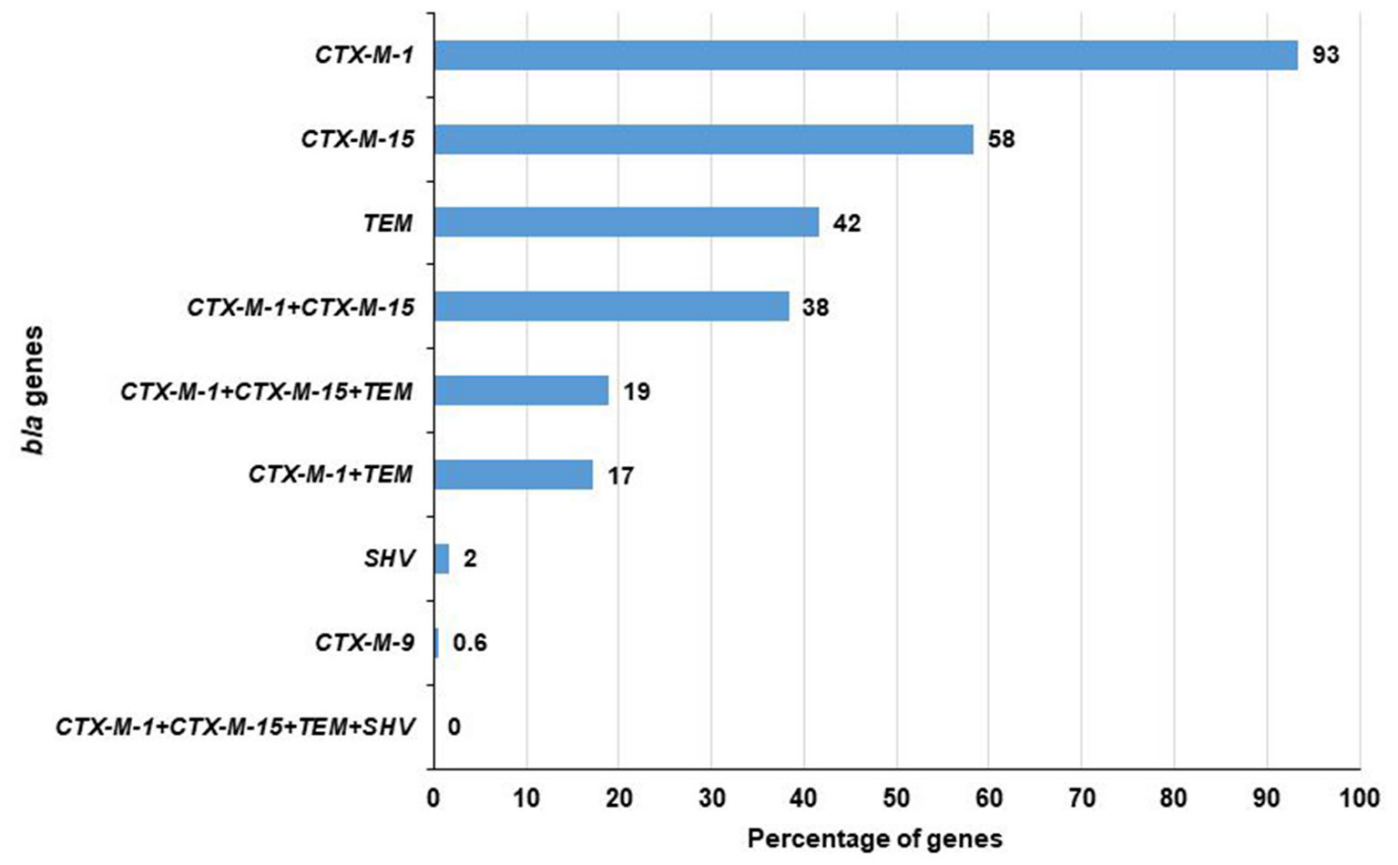
Prevalence of different *bla* genes in ESBL producing *E. coli*.

Among 180 ESBL-*E. coli*, 179 contained at least one of the tested ESBL genes i.e., *bla*_*CTX*−*M*−1_, *bla*_*CTX*−*M*−2_, *bla*_*CTX*−*M*−8_, *bla*_*CTX*−*M*−9_, *bla*_*CTX*−*M*−15_, *bla*_*CTX*−*M*−25_, *bla*_*TEM*_, and *bla*_*SHV*_. A total of 93% (168/180) isolates were positive for *bla*_*CTX*−*M*−1_ gene followed by 58% (105/180) for *bla*_*CTX*−*M*−15_, 42% (75/180) for *bla*_*TEM*_, 2% (3/180) for *bla*_*SHV*_ and 0.6% (1/180) for *bla*_*CTX*−*M*−9_ genes. No isolate carried *bla*_*CTX*−*M*−2_, *bla*_*CTX*−*M*−8_, and *bla*_*CTX*−*M*−25_ genes. Notably, among the *bla*_*CTX*−*M*−1_, *bla*_*CTX*−*M*−15_, *bla*_*TEM*_, and *bla*_*SHV*_ genes, a maximum of three genes (*bla*_*CTX*−*M*−1_, *bla*_*CTX*−*M*−15_, and *bla*_*TEM*_) were found in 19% (34/180) of the isolates. In addition, *bla*_*CTX*−*M*−1_ and *bla*_*CTX*−*M*−15_ were found to co-exist in 38% (69/180) and *bla*_*CTX*−*M*−1_ and *bla*_*TEM*_ in 17% (31/180) of the isolates. No isolate was found to carry these four genes together (Figure 2). In case of the inlet, *bla*_*CTX*−*M*−1_ was present in 93% (70/75) of isolates followed by *bla*_*CTX*−*M*−15_ in 57% (43/75), *bla*_*TEM*_ in 49% (37/75), and *bla*_*SHV*_ in 1% (1/75) of the isolates. However, in the outlet, it was 93% (98/105), 59% (62/105), 36% (38/105), and 2% (2/105), respectively (Table 1).

**Table 1:** Presence of *bla* genes in ESBL positive *E. coli* isolates detected *via* PCR.

	***bla***_***CTX*** **−** ***M*** **−1**_		***bla***_***CTX*** **−** ***M*** **−9**_		***bla***_***CTX*** **−** ***M*** **−15**_		***bla***_***TEM***_		***bla***_***SHV***_	
**Total isolates**	**93% (168/180)**		**0.6% (1/180)**		**58% (105/180)**		**42% (75/180)**		**2% (3/180)**	
	**Inlet**	**Outlet**	**Inlet**	**Outlet**	**Inlet**	**Outlet**	**Inlet**	**Outlet**	**Inlet**	**Outlet**
Number of isolates	70	98	1	0	43	62	37	38	1	2
Percentage	93% (70/75)	93% (98/105)	1.33% (1/75)	0%	57% (43/75)	59% (62/105)	49% (37/75)	36% (38/105)	1% (1/75)	2% (2/105)

### High Prevalence of Plasmid Containing Isolates

**Figure 3:**
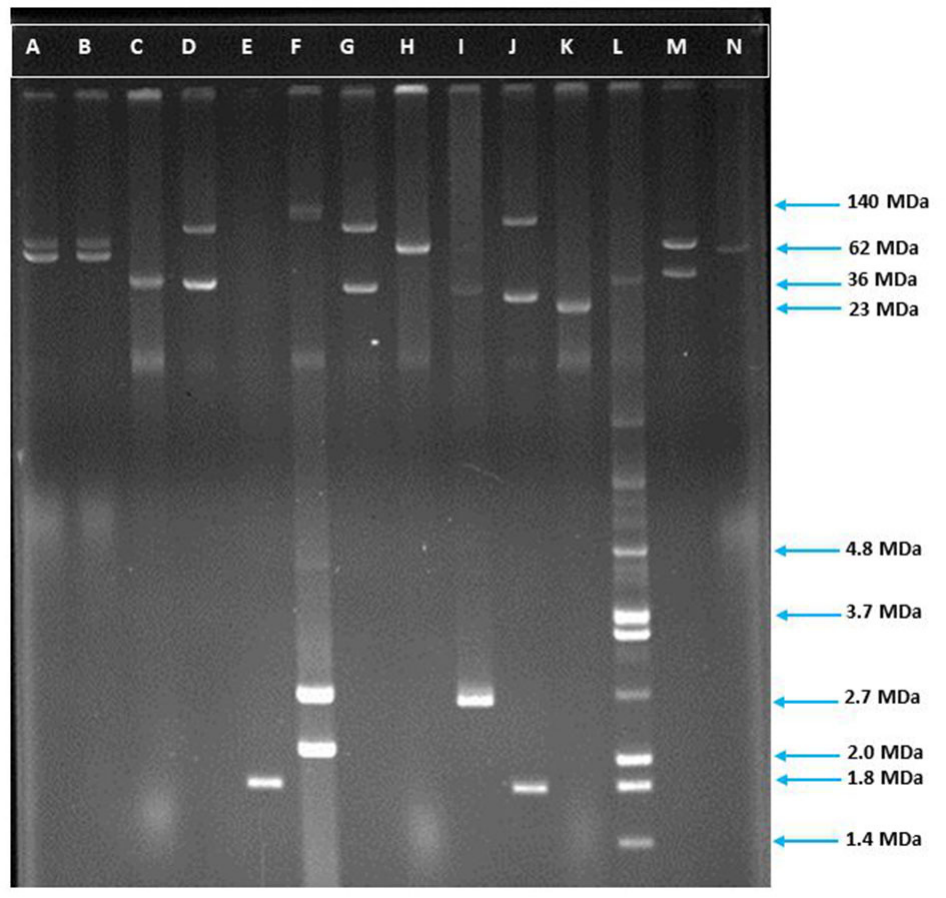
Agarose gel electrophoresis of plasmid DNA showing the patterns among the ESBL positive isolates. Lane-C: RP4 (36 MDa), Lane-F: *E. coli* strain PDK9 (140, 105, 2.7, 2.1 MDa), Lane-H: R1 (62 MDa), Lane-K: Sa (23 MDa), Lane-L: V517 (35.8, 3.4, 3.7, 2.0, 1.8, 1.4), Lane-A, B, D, E, G, I, J, M, and N are ESBL positive *E. coli*. The molecular weight of the markers is shown in the picture.

**Figure 4:**
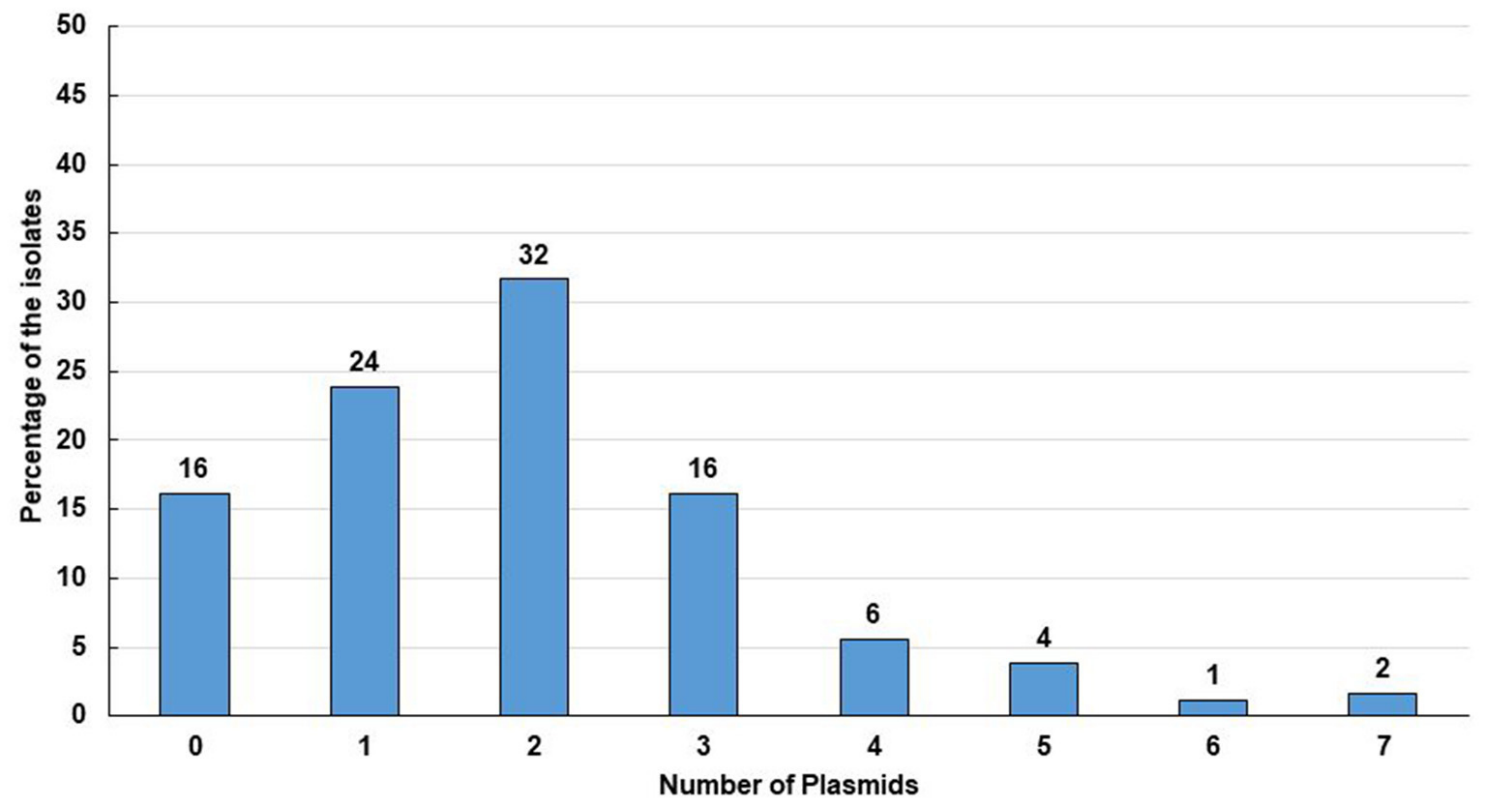
Percentage of isolates with number of plasmids they contained. Tweenty nine isolates contained no plasmid which comprises 16% of the total isolates. Accordingly, 43 (24%) of the isolates contained a single plasmid, 57 (32%) isolates contained double plasmids, 29 (16%) isolates contained 3 plasmids, 10 (6%) isolates contained 4 plasmids, 7 (4%) isolates contained 5 plasmids, 2 (1%) isolates contained 6 plasmids and 3 (2%) isolates contained 7 plasmids.

Plasmids play a significant role in antimicrobial resistance, virulence, microbial adaptation, and evolution. Moreover, antibiotic resistance genes are mostly plasmid coded (7, 8). We, therefore, determined the plasmid number and size in ESBL positive *E. coli* isolates. Plasmid analysis showed that 84% (151/180) of the isolates contained plasmids of different sizes ranging from 1.19 to 211.54 MDa (Figures 3, 4). Among them, 72% (108/151) plasmid containing isolates possessed more than one plasmid, whereas 28% (43/151) owned only a single plasmid. Maximum seven plasmids were found in three isolates only (Supplementary Table 4).

### Both Diarrhoeagenic and Extraintestinal Pathogenic *E. coli* Were Found in Faecal Sludge

**Figure 5:**
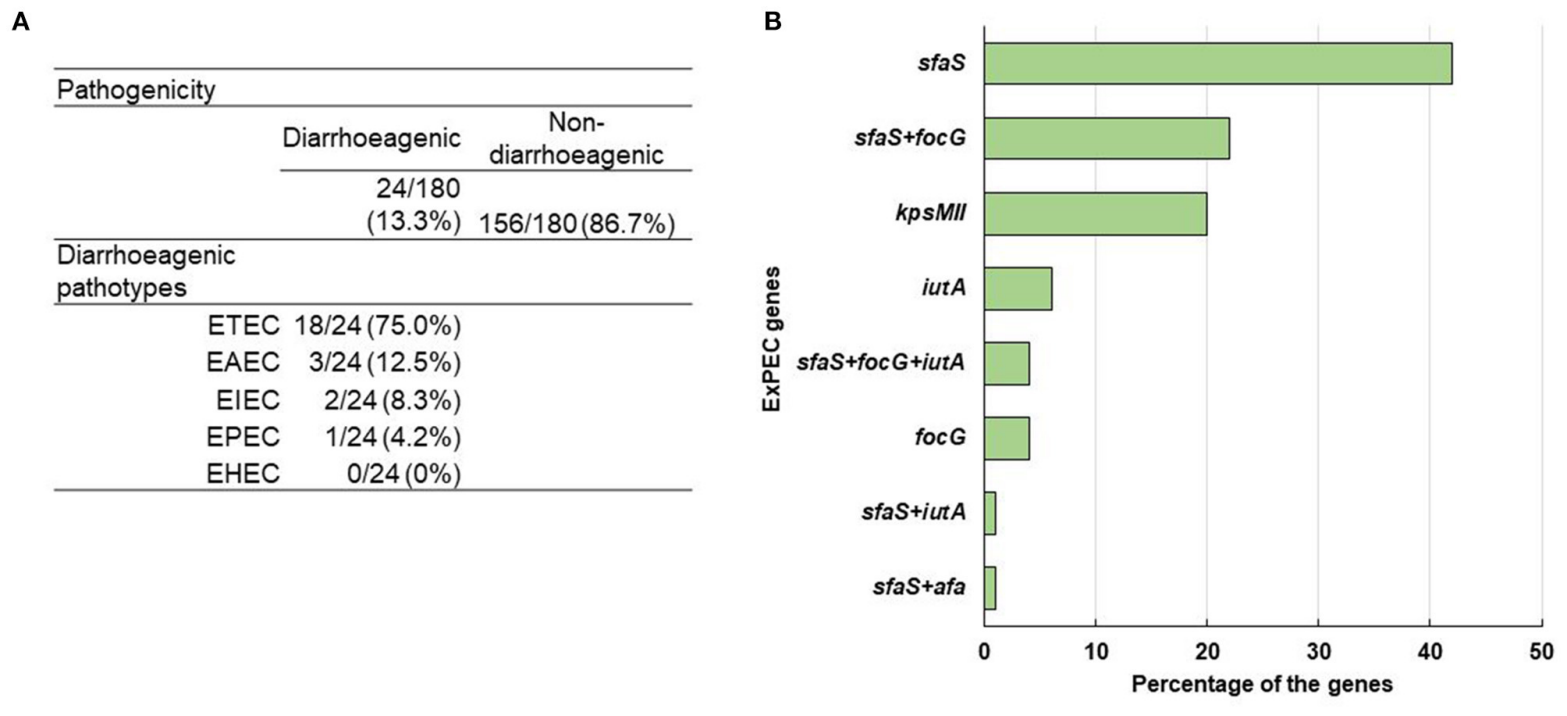
**(A)** Distribution of diarrheagenic pathogens and different *E. coli* pathotypes. **(B)** Percentage of ExPEC genes among the positive isolates.

In the multiplex PCR, for the detection of ETEC, EPEC, EAEC, EHEC and EIEC, a well-characterized ETEC (for *lt, st*), EPEC (for *bfp, eae*), EAEC (for *aat, aaiC*), EHEC (for *stx 1, stx 2)*, and EIEC K-309 (for *ipaH, ial*) strains were used as positive controls. At least one diarrhoeagenic virulence gene was detected in 13% (24/180) of the ESBL producing *E. coli* isolates among the ten tested *E. coli* pathotype-specific virulence genes. Among them, two-thirds were from the outlet and the rest were from the inlet. Besides, 12 and 5 isolates were found positive for only *lt* and *st* genes, respectively, whereas only 1 was positive for both *lt* and *st* genes. However, 3 isolates were positive for *aat*, and 1 isolate for *bfp, ial*, and *ipaH* gene each. None of the isolates was found positive for *eae, aaiC, stx 1*, and *stx 2* genes. So, among the 24 diarrhoeagenic *E. coli* isolates, 75% (18/24), 13% (3/24), 8% (2/24), and 4% (1/24) were found positive for ETEC, EAEC, EIEC, and EPEC, respectively (Figure 5A).

In the case of ExPEC virulence factor, five out of seven genes were detected that comprised *sfaS, focG, kpsMII, iutA*, and *afa* whereas their prevalence rates were 35% (55/156), 15% (23/156), 10% (16/156), 5% (8/156), and 1% (1/156), respectively. Among 156 non-diarrhoeagenic isolates, 51% (79/156) were positive for at least one ExPEC virulence factor. From those, 42% (33/79), 20% (16/79), 6% (5/79), and 4% (3/79) were positive for only *sfaS, kpsMII, iutA*, and *focG*, respectively. Though both *sfaS* + *focG* were present in 17 isolates, *sfaS* + *afa* and *sfaS* + *iutA* were in 1 isolate each. Interestingly, 4% (3/79) isolates were found to carry *sfaS*+ *focG*+ *iutA* genes (Figure 5B). Though 79 isolates had at least one ExPEC virulence marker, 3 isolates were identified as ExPEC strains because of harboring three or more ExPEC associated genes (52).

### Pathogenic *E. coli* Isolates Were Multidrug-Resistant

**Figure 6:**
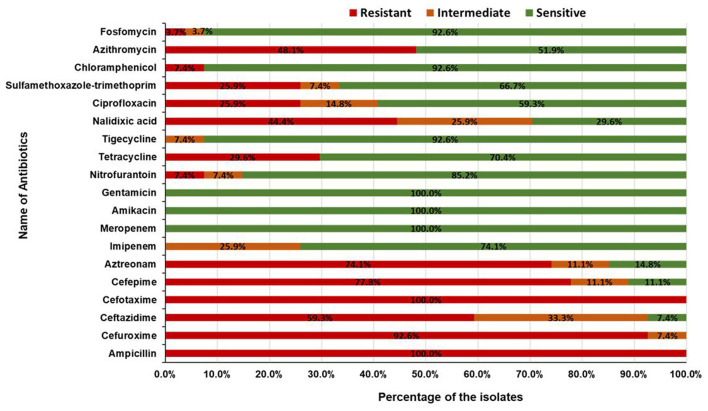
Antibiotic resistance patterns of pathogenic ESBL producing *E. coli* isolates.

To explore the multidrug resistance properties of the pathogenic isolates, antibiotic resistance profiles of 24 diarrhoeagenic and 3 ExPEC isolates were determined by the Kirby-Bauer disk diffusion method. A total of 19 antimicrobial agents from 15 different antibiotic classes i.e. penicillin, cephalosporin (second, third, and fourth generation), monobactam, carbapenem, aminoglycoside, nitrofuran, tetracycline, glycylcycline, quinolone, sulfonamide, phenicol, macrolides, and phosphonic acid were used. All of the 27 isolates were resistant to ampicillin and cefotaxime. About 78% (21/27) of the isolates were found to be resistant to cefuroxime, followed by 59% (16/27) to ceftazidime, 78% (21/27) to cefepime, 74% (20/27) to aztreonam, 7% (2/27) to nitrofurantoin, 30% (8/27) to tetracycline, 44% (12/27) to nalidixic acid, 26% (7/27) to ciprofloxacin, 26% (7/27) to sulfamethoxazole-trimethoprim, 7% (2/27) to chloramphenicol, 48% (13/27) to azithromycin, and 4% (1/27) to fosfomycin. None of the isolates was found resistant to gentamicin, imipenem, meropenem, amikacin, and tigecycline (Figure 6). Surprisingly, all of the pathogenic isolates were multidrug-resistant and 41% (11/27) of isolates were resistant to seven or more classes of antibiotics (Table 2). The interpretation based on the diameter of zone of inhibition is given in Supplementary Table 2.

**Table 2:** Number of isolates resistant to different classes of antibiotics.

**Classes of antibiotics**	**Number of isolates**
Penicillin + 2G Cephalosporin + 3G Cephalosporin + 4G Cephalosporin	1
Penicillin + 2G Cephalosporin + 3G Cephalosporin + 4G Cephalosporin + Monobactam	5
Penicillin + 2G Cephalosporin + 3G Cephalosporin + 4G Cephalosporin + Monobactam + Macrolide	2
Penicillin + 2G Cephalosporin + 3G Cephalosporin + 4G Cephalosporin + Monobactam + Nitrofuran + Quinolone	1
Penicillin + 2G Cephalosporin + 3G Cephalosporin + 4G Cephalosporin + Monobactam + Nitrofuran + Quinolone + Macrolide	1
Penicillin + 2G Cephalosporin + 3G Cephalosporin + 4G Cephalosporin + Monobactam + Quinolone	1
Penicillin + 2G Cephalosporin + 3G Cephalosporin + 4G Cephalosporin + Monobactam + Quinolone + Sulfonamide + Macrolide	1
Penicillin + 2G Cephalosporin + 3G Cephalosporin + 4G Cephalosporin + Monobactam + Quinolone + Sulfonamide + Phenicol + Phosphonic acid	1
Penicillin + 2G Cephalosporin + 3G Cephalosporin + 4G Cephalosporin + Monobactam + Sulfonamide	1
Penicillin + 2G Cephalosporin + 3G Cephalosporin + 4G Cephalosporin + Monobactam + Sulfonamide + Macrolide	2
Penicillin + 2G Cephalosporin + 3G Cephalosporin + 4G Cephalosporin + Monobactam + Tetracycline + Macrolide	3
Penicillin + 2G Cephalosporin + 3G Cephalosporin + 4G Cephalosporin + Monobactam + Tetracycline + Quinolone	1
Penicillin + 2G Cephalosporin + 3G Cephalosporin + 4G Cephalosporin + Monobactam + Tetracycline + Quinolone + Phenicol	1
Penicillin + 2G Cephalosporin + 3G Cephalosporin + Quinolone + Sulfonamide + Macrolide	1
Penicillin + 2G Cephalosporin + 3G Cephalosporin + Tetracycline + Quinolone	2
Penicillin + 2G Cephalosporin + 3G Cephalosporin + Tetracycline + Quinolone + Macrolide	1
Penicillin + 3G Cephalosporin + Macrolide	1
Penicillin + 3G Cephalosporin + Quinolone + Sulfonamide + Macrolide	1
**Total**	**27**

### Genetic Fingerprinting of the Pathogenic ESBL *E. coli*

**Figure 7:**
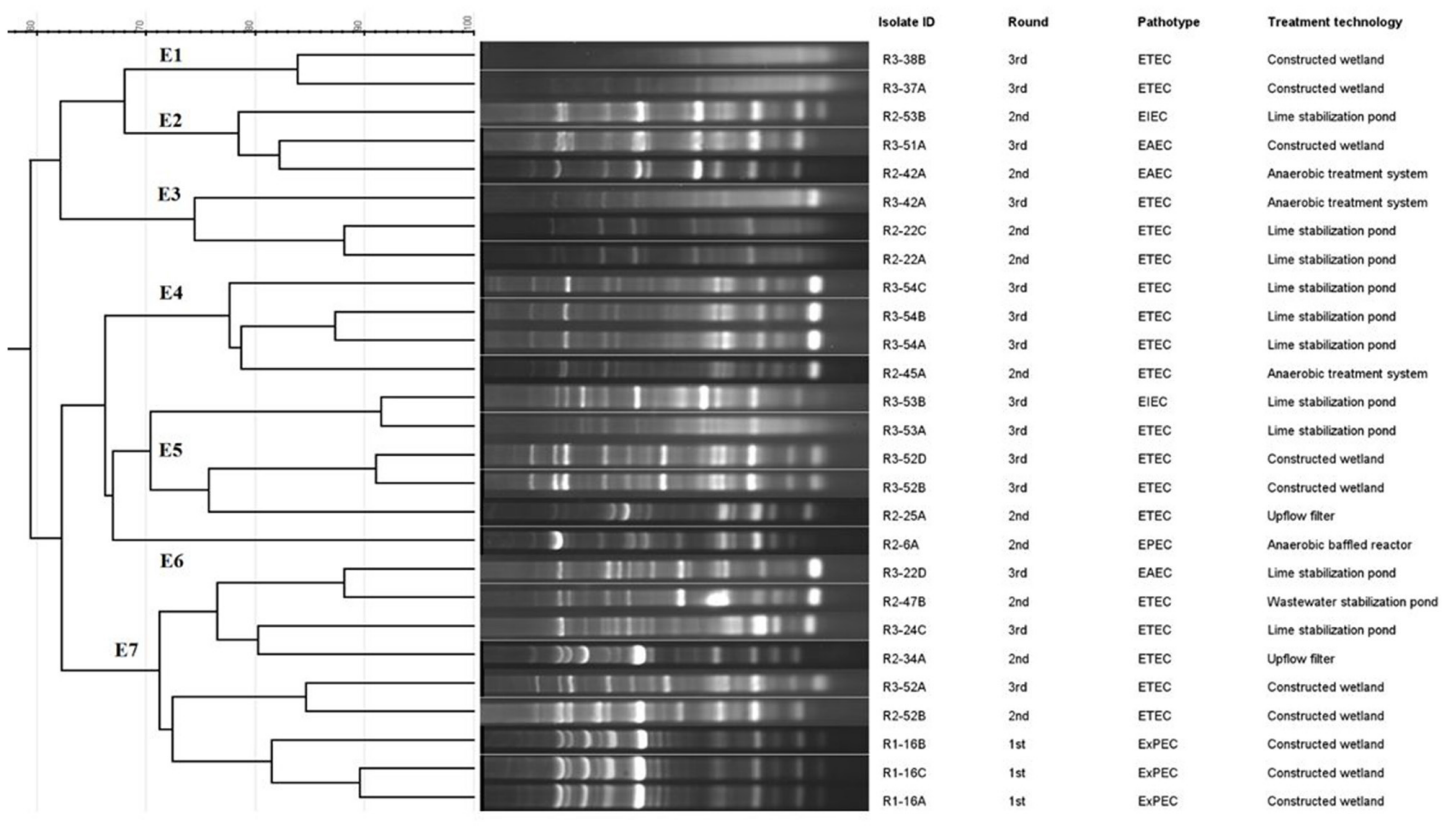
Dendrogram of ERIC-PCR fingerprints from the pathogenic *E. coli* isolates. The percentage of genetic homology among banding patterns is indicated. Isolate ID, round, pathotypes and treatment technology of the plants are plotted next to dendrogram.

We investigated the clonal relatedness of the ESBL producing pathogenic *E. coli* by ERIC-PCR for molecular typing to calculate the visibility and placement of the gels according to their molecular weights and molecular markers. The genotyping profiles of 27 *E. coli* isolates according to ERIC-PCR fingerprinting are shown in Figure 7. Dendrogram analysis revealed that ERIC-PCR differentiated the isolates into seven clusters, E1–E7 with 70% similarity. The isolates produced 4–17 amplicons ranging from 180 to 2,000 bp, where 200, 320, and 1,250 bp were common in most of the isolates. Here the maximum 9 isolates were in E7, 5 in E5, 4 in E4, 3 in both E2 and E3, 2 in E1 and a single isolate in the E6 cluster.

### A Significant Proportion of Isolates Transferred Their Plasmids *via* Conjugation

We explored the ARGs transferability of the ESBL producing *E. coli via* conjugation using *E. coli*-J53 plasmid recipient strain. Conjugation experiments using representative isolates (*n* = 35) with diverse plasmid patterns demonstrated that plasmids ranging in size from 32.13 to 112.20 MDa were self-transmissible to a sodium azide resistant *E. coli*-J53 recipient strain but the smaller plasmids (<30 MDa) could not be transferred. With the transfer rates ranging from 4.3 × 10^−9^ to 1.82 × 10^−4^ per donor cell, 16 isolates successfully transferred the cefotaxime resistance determinants to a susceptible *E. coli* recipient (Table 3). Analysis of antibiotic susceptibility patterns of transconjugants showed that cefuroxime, tetracycline, ceftazidime, cefepime, aztreonam, nalidixic acid, sulfamethoxazole-trimethoprim, ciprofloxacin, and azithromycin resistance were co-transferred through plasmids along with cefotaxime resistance. On the other hand, phenicol resistance genes were not transferred *via* conjugative plasmids. Among the 16 donors, maximum of 3 plasmids were transferred by a single isolate whereas a minimum of 1 plasmid was transferred by 7 isolates each.

**Table 3:** Findings of conjugation assays between antibiotic resistant *E. coli* isolates obtained from faecal sludge samples and the recipient *E. coli* J53 strain.

**Isolate ID**	**Parent strain**			**Transconjugant**			**Transfer rate**
	**Plasmid pattern**	**ESBL gene pattern**	**Resistance pattern**	**Plasmid pattern**	**ESBL gene pattern**	**Resistance pattern**	
R1-1A	65.80; 54.33	CTXM-1, CTXM-15	AMP, CXM, CAZ, CTX, FEP, ATM, TE, NA, SXT	54.33	CTXM-1, CTXM-15	AMP, CXM, CAZ, CTX, FEP, ATM	3.47 × 10^−6^
R2-6A	94.84; 67.56	CTXM-1, TEM	AMP, CXM, CAZ, CTX, FEP, ATM, AZM	67.56	CTXM-1, TEM	AMP, CXM, CAZ, CTX, FEP, ATM, AZM	7.79 × 10^−9^
R2-22A	66.45; 56.02; 2.87	CTXM-1	AMP, CXM, CAZ, CTX, FEP, ATM, TE, AZM	66.45; 56.02	CTXM-1	AMP, CXM, CAZ, CTX, FEP, ATM, TE, AZM	4.3 × 10^−9^
R2-52B	81.91; 1.58	CTXM-1, CTXM-15	AMP, CXM, CAZ, CTX, FEP, ATM, TE, NA, CIP	81.91	CTXM-1, CTXM-15	AMP, CXM, CAZ, CTX, FEP, ATM, TE, NA, CIP	2.84 × 10^−8^
R3-15A	86.25; 37.24	CTXM-1, CTXM-15, TEM	AMP, CXM, CAZ, CTX, FEP, ATM	86.25; 37.24	CTXM-1, CTXM-15	AMP, CXM, CAZ, CTX, FEP, ATM	3.73 × 10^−8^
R3-29B	87.39; 32.13	CTXM-1, CTXM-15, TEM	AMP, CXM, CAZ, CTX, FEP, SXT	87.39; 32.13	CTXM-1, CTXM-15, TEM	AMP, CXM, CTX, FEP, SXT	2.57 × 10^−7^
R3-37A	112.20; 61.11; 52.68	CTXM-1, CTXM-15	AMP, CXM, CTX, FEP, ATM, F, NA, AZM	112.20; 61.11; 52.68	CTXM-1, CTXM-15	AMP, CXM, CTX, FEP, ATM, AZM	1.27 × 10^−8^
R3-42A	110.56; 64.61; 4.82; 2.50	CTXM-1, CTXM-15	AMP, CXM, CAZ, CTX, FEP, ATM, TE, NA, CIP, C	64.61	CTXM-1, CTXM-15	AMP, CXM, CAZ, CTX, FEP, ATM	4.29 × 10^−8^
R3-45B	78.51; 61.08; 49.40; 6.47	CTXM-1, CTXM-15, TEM	AMP, CXM, CAZ, CTX, FEP, ATM, NA	61.08	CTXM-1, CTXM-15	AMP, CXM, CAZ, CTX, FEP, ATM	1.82 × 10^−4^
R3-49A	75.78; 38.63	CTXM-1	AMP, CXM, CAZ, CTX, FEP, ATM	75.78; 38.63	CTXM-1	AMP, CXM, CAZ, CTX, FEP, ATM	1.96 × 10^−6^
R3-52A	71.39; 48.69	CTXM-1, CTXM-15	AMP, CXM, CTX, FEP, ATM	71.39; 48.69	CTXM-1, CTXM-15	AMP, CXM, CTX, FEP, ATM	7.5 × 10^−8^
R3-52B	71.39; 48.69	CTXM-1, CTXM-15	AMP, CXM, CAZ, CTX, FEP, ATM, SXT	71.39; 48.69	CTXM-1, CTXM-15	AMP, CXM, CAZ, CTX, FEP, ATM	8.82 × 10^−8^
R3-52D	71.39; 48.69	CTXM-1, CTXM-15	AMP, CXM, CAZ, CTX, FEP, ATM	71.39; 48.69	CTXM-1, CTXM-15	AMP, CXM, CAZ, CTX, FEP, ATM	1.62 × 10^−8^
R3-53A	77.07; 59.55	CTXM-1, CTXM-15	AMP, CXM, CTX, FEP, ATM, SXT, AZM	77.07	CTXM-1, CTXM-15	AMP, CXM, CTX, ATM	1.43 × 10^−4^
R3-53B	77.07; 59.55	CTXM-1, CTXM-15	AMP, CXM, CAZ, CTX, FEP, ATM, SXT, AZM	77.07	CTXM-1, CTXM-15	AMP, CXM, CTX	1.52 × 10^−4^
R3-54B	57.51; 39.24	CTXM-1, CTXM-15	AMP, CXM, CAZ, CTX, FEP, ATM	57.51; 39.24	CTXM-1, CTXM-15	AMP, CXM, CAZ, CTX, FEP, ATM	5.10 × 10^−9^

## Discussion

Antimicrobial resistance has expanded globally, posing a risk to the efficient treatment of various infectious diseases (57, 58). The rise of such resistance has been associated with faecal contamination of surface water, recreational water, marshlands and even potable water (59–63). In addition, pathogenic bacteria are 10–100 times more abundant in faecal sludge than in wastewater, posing environmental, and health risks (64). So, there is every possibility of occurring MDR pathogenic bacteria in faecal sludge and infections with them limit therapeutic options.

This study was aimed to investigate the ability of *E. coli* to produce ESBLs, the presence of virulence factors, antibiotic resistance patterns and plasmid transferability. In this study, among a total of 108 FSTP samples, 100% (54/54) samples from the inlets and 85% (46/54) samples from the outlets were contaminated with *E. coli*. In our study, ESBL producing *E. coli* was recovered from 78% (42/54) of the inlet and 69% (37/54) of the outlet samples. Previously, ESBL producing *E. coli* were recovered from faecal samples (41, 65–68) and sewage sludge (69, 70). Recovery of ESBL *E. coli* from the samples manifested a real probability of bacterial circulation within the FSTPs and might also escape to the surrounding environment and eventually contaminate the surface water and groundwater.

In this study, among the 180 ESBL producers, 179 were positive for β-lactamases of class A having clinical significance. Among the ARGs, *bla*_*CTX*−*M*−1_ and *bla*_*CTX*−*M*−15_ were found 93% (168/180) and 58% (105/180) of the isolates, respectively. Interestingly, in our study, 42% (75/180) contained *bla*_*TEM*_, while 2% (3/180) were positive for *bla*_*SHV*_. It has been reported before that genotypic characterization showed a dominance of the *bla*_*CTX*−*M*−1_ group similar to our study (71). *E. coli* strains expressing *bla*_*CTX*−*M*−15_ have arisen and spread around the world and are currently a major causative agent of hospital-acquired and community-onset urinary tract, and bloodstream infection in humans (72, 73). However, *bla*_*CTX*−*M*−15_ seems to be the most extensive type in isolates of human origin (74). Our findings pointed out the importance of tracking and monitoring clinically significant *bla*_*TEM*_ gene and *bla*_*CTX*−*M*_ gene-positive strains and signifying the importance of finding a solution for it.

The study of plasmids facilitates understanding their role in human and animal health, environmental processes, and microbiological adaptation and evolution. Plasmids can encode genes responsible for resistance or virulence emphasizing its importance in biomedical research (44). In this study, plasmid profiling was performed for all the 180 ESBL producing *E. coli* isolates as most of the ESBLs are primarily plasmid-coded and can disseminate through horizontal gene transfer between the same bacteria and even between different species (8). In addition to this, resistance to antibacterial agents, other than β-lactams, may also be located on these plasmids at the same time and can contribute to the dissemination of resistance (75). The gene transfer process is critical in faecal sludge due to the higher density of microorganisms and more frequent contact between them which further facilitates the elevated rate of gene exchange. Previously, it has been reported that plasmids harboring the ESBL gene may contain several β-lactamase genes, for example, CTX-M and TEM (76), and CTX-M was found to be the most prominent among plasmid-coded β-lactamases (69). According to the plasmid profile analysis, the majority, 60% (108/180) of the isolates carried multiple plasmids and minimal similarity among the plasmid patterns of the isolates pointed to their clonal diversity. The more plasmids a bacteria contains, the greater antibiotic resistance is likely to have. It was previously shown that containing multiple plasmids simultaneously conferred co-resistance to antimicrobial agents of different groups (77). The current study also disclosed that a significant number of isolates, 67% (120/180), contained large plasmids ranging from 50 to 211 MDa. Plasmids of these sizes have already been reported to be self-transmissible which eventually transfer the antimicrobial resistance determinants to *Enterobacteriaceae*, remarkably to *Shigella* spp. and *E. coli* (56, 78, 79). Additionally, it has been reported that plasmids of >120 MDa possess invasive characteristics for specific gastrointestinal pathogens, like *Shigella* spp., and EIEC (80). In our study, 3 isolates possessed plasmids of that size. Another concern is that conjugative transfer of plasmid coded ESBL genes has been observed in 16 isolates out of 35 in our study based on plasmid sizes. Cefotaxime resistance carrying conjugative plasmids co-transferred several other antibiotic resistance with different transfer frequencies. These outcomes suggest that horizontal gene transfer could exacerbate the current antibiotic resistance situation by accelerating AMR in environmentally heterogeneous bacterial communities (81). The conjugal translocation of resistance plasmids was unsuccessful for a number of isolates. In these isolates, the resistance gene may be located in plasmids that are non-conjugative or chromosomally encoded. To figure out where the resistance genes reside in these isolates, extensive research is required.

In places where enteric infections are endemic, pathogenic *E. coli* promotes the prevalence of infectious diseases significantly. In this study, 24 out of 180 isolates were identified as diarrhoeagenic pathogens from faecal sludge samples. The predominant pathotype was ETEC, comprising 75% (18/24) of the diarrhoeagenic isolates and this finding is in agreement with previous studies from human pit sludge and stool samples (41, 82). ETEC is responsible for almost 20% of all cases of diarrhea in children under the age of two (83). This pathotype has already been found to be prevalent in drinking and environmental water, and alive after long-standing incubation of water, implying that water could be a major mode of transmission (84, 85). Other than ETEC, we also found EAEC, EIEC, and EPEC accountable for 13% (3/24), 8% (2/24), and 4% (1/24) of the pathogenic isolates, respectively, and interestingly all of them are ESBL positive. Though it is still unclear, the possible reservoir of EAEC is widely assumed to be human (86–88). It is suspected that EAEC adopts the faecal-oral route for transmission as it is often reported as waterborne or foodborne (89). A high ratio of ESBL in EAEC has also been reported in recent studies from Iran and China (90, 91). In addition to the diarrhoeagenic *E. coli*, 4% (3/156) of the isolates have been characterized as ExPEC strains. So, inappropriately treated faecal sludges might result in impending epidemics, like diarrhoea and other extraintestinal diseases (e.g., UTI, meningitis), which necessitates the proper treatment of faecal sludge before exposure to the environment.

Antibiotic susceptibility profile analysis revealed that all the pathogenic isolates were multidrug-resistant. The increased ratio of multidrug-resistant *E. coli* among ESBL positive isolates suggests that resistance to other antibiotic classes is being co-selected. Similarly, previous studies reported MDR organisms in faecal and sewage sludge samples (41, 65, 66, 68, 70). The most frequently used antibiotics in Bangladesh are cephalosporins and penicillins (92), which explains the reason for the isolates being resistant to cephalosporins and penicillins. Surprisingly, a significant number of isolates, 77.8% (21/27), remained resistant to cefepime, a fourth-generation cephalosporin. Furthermore, a high percentage of them has shown resistance to the quinolone class. This could be due to the excessive use or misuse of antibiotics which are commonly sold and distributed across the country (93, 94). We also observed that 100% (27/27) of the isolates were sensitive to one of the carbapenems (meropenem) and aminoglycoside (amikacin, gentamicin) groups. On the contrary, 26% (7/27) of the isolates were found intermediate to imipenem, which indicates a fear of being resistant to that antibiotic shortly if awareness is not raised immediately.

In the present study, genetic fingerprint patterns have been explored for 27 ESBL producing pathogenic *E. coli* isolates using ERIC-PCR. The constructed dendrogram has grouped the isolates into 7 clusters at 70% similarity. The genetic association was demonstrated in the cluster where most isolates are distributed, though they were isolated from different FSTPs at different times. Nine pathogenic isolates clustered together despite having differences in their pathotypes, collection time and sites suggesting their genetic linkage. This finding is similar to the previous reports where genetically diverse *E. coli* was found in the environment (95–97). This genetic variation could be due to the mutation when they persist in the host and the environment (98).

The present study was limited to only three sampling rounds, thus extensive investigation is required for a better understanding of the actual scenario. Moreover, we could not conduct any experiment to know the migration of ESBL- *E. coli* and transmission of resistance genes to the surrounding environment. Whole-genome sequencing of the pathogenic MDR ESBL *E. coli* isolates would enlighten the mechanism of antibiotic resistance and transmission dynamics.

We found plasmid containing ESBL *E. coli* in most of the samples and confirmed that all the pathogenic *E. coli* to be multidrug-resistant. High prevalence of MDR pathogenic commensal *E. coli* is associated with a higher risk of infections, higher costs, longer hospital stays, prolonged stays in ICU, severe primary disease and frequent administration of antibiotics etc. Improper handling and disposal of the effluents and sludge of treatment plants into inappropriate sites may also compromise public health by contaminating the land, water supplies, or recreational waters, and facilitating the spread of microorganisms and resistance genes into the environment. All of this evidence is highly concerning because the horizontal transfer of resistance genes may worsen the situation and eventually outbreaks may origiante from environmental samples or contaminated food and water sources. Quick actions are needed to restrict the development of resistance in the environment. For preventative reasons, optimum treatment should be given preference considering the degree of resistant pathogens introduction into the environment. Future research should be conducted to establish a standardized approach for detecting ESBL *E. coli* in faecal sludge. Unnecessary antibiotic therapies must be avoided, and unregulated therapies should be limited. Effective control measures are strongly suggested for the protection of public health to prevent pathogenic *E. coli* contamination of food and water bodies coming from faecal sludge.

## Data Availability Statement

The original contributions presented in the study are included in the article/Supplementary Material, further inquiries can be directed to the corresponding author.

## Ethics Statement

The Ethical Review Committee of International Center for Diarrhoeal Disease Research, Bangladesh has approved the study.

## Author Contributions

ZM, SA, and MSH: conceptualization. MSH, SA, SU, and MH: methodology. MSH and SA: software. ZM, AS, MSI, MM, SA, SU, MH, and MRI: validation and investigation. MSH, SA, MH, and MM: formal analysis and data curation. ZM and MSI: resources. MSH: writing—original draft preparation. ZM, MSH, SA, MM, MH, SU, MRI, MSI, TA, MAR, MW, and DM: writing—review and editing. ZM: supervision, project administration, and funding acquisition. All authors have read and agreed to the published version of the manuscript.

## Funding

This research study was funded by United Nations Children's Fund (unicef), grant number 43252528. International Centre for Diarrhoeal Disease Research, Bangladesh (icddr,b) acknowledges with gratitude the commitment of unicef to its research efforts. icddr,b is also grateful to the Governments of Bangladesh, Canada, Sweden, and the UK for providing unrestricted support.

## Conflict of Interest

The authors declare that the research was conducted in the absence of any commercial or financial relationships that could be construed as a potential conflict of interest.

## Publisher's Note

All claims expressed in this article are solely those of the authors and do not necessarily represent those of their affiliated organizations, or those of the publisher, the editors and the reviewers. Any product that may be evaluated in this article, or claim that may be made by its manufacturer, is not guaranteed or endorsed by the publisher.
