# Supplementary material for: ESBL Producing Escherichia coli in Faecal Sludge Treatment Plants: An Invisible Threat to Public Health in Rohingya Camps, Cox's Bazar, Bangladesh
Source: Front Public Health. 2021 Dec 15;9:783019. doi: 10.3389/fpubh.2021.783019 (PMC8714839; doi:10.3389/fpubh.2021.783019)
Supplement: Supplementary file 1 [file Table_1.DOCX]

**Supplementary Table 1.** List of primers used in this study

|  | **Target gene** | **Primer name** | **Sequence (5’ 🡪 3’)** | **Product size (bp)** | **Reference** |
| --- | --- | --- | --- | --- | --- |
| **ESBL primers** | *bla_CTX-M-1_* | M13-upper | GGTTAAAAAATCACTGCGTC | 866 | (36) |
|  |  | M13-lower | TTGGTGACGATTTTAGCCGC |  |  |
|  | *bla_CTX-M-2_* | M25-upper | ATGATGACTCAGAGCATTCG | 866 |  |
|  |  | M25-lower | TGGGTT ACGATTTTCGCCGC |  |  |
|  | *bla_CTX-M-8_* | CTXM8 -F | TCGCGTTAAGCGGATGATGC | 688 | (37) |
|  |  | CTXM8-R | AACCCACGATGTGGGTAGC |  |  |
|  | *bla_CTX-M-9_* | M9-upper | ATGGTGACAAAGAGAGTGCA | 870 | (36) |
|  |  | M9-lower | CCCTTCGGCGATGATTCTC |  |  |
|  | *bla_CTX-M-15_* | CTX-M-15-SF | CACACGTGGAATTTAGGGACT | 996 | (38) |
|  |  | CTX-M-15-SR | GCCGTCTAAGGCGATAAACA |  |  |
|  | *bla_CTX-M-25_* | CTX-M-25-F | CACACGAATTGAATGTTCAG | 924 | (39) |
|  |  | CTX-M-25-R | TCACTCCACATGGTGAGT |  |  |
|  | *bla_SHV_* | SHV-F | CACTCAAGGATGTATTGTG | 885 | (41) |
|  |  | SHV-R | TTAGCGTTGCCAGTGCTCG |  |  |
|  | *bla_TEM_* | TEM-F | TCGGGGAAATGTGCGCG | 850 | (40) |
|  |  | TEM-R | TGCTTAATCAGTGAGGACCC |  |  |
| **Intestinal pathogenic primers** | *estA* | ST-F | GCTAAACCAGTA^G^_A_GGTCTTCAAAA | 147 | (99) |
|  |  | ST-R | CCCGGTACA^G^_A_GCAGGATTACAACA |  |  |
|  | *eltB* | LT-F | CACACGGAGCTCCTCAGT C | 508 | (40) |
|  |  | LT-R | CCCCCAGCCTAGCTTAGTTT |  |  |
|  | *bfpA* | bfpA-F | GGAAGTCAAATTCATGGGGG | 300 | (40) |
|  |  | bfpA-R | GGAATCAGACGCAGACTGGT |  |  |
|  | *eae* | eae-F | CCCGAATTCGGCACAAGCATAAGC | 881 | (100) |
|  |  | eae-R | CCCGGATCCGTCTCGCCAGTATTCG |  |  |
|  | *aaiC* | aaiC-F | ATTGTCCTCAGGCATTTCAC | 215 | (40) |
|  |  | aaiC-R | ACGACACCCCTGATAAACAA |  |  |
|  | *aat* | _p_cvd432-F | CTGGCGAAAGACTGTATCAT | 650 | (101) |
|  |  | _p_cvd432-R | CAATGTATAGAAATCCGCTGTT |  |  |
|  | *iaa* | ial upper | CTGGATGGTATGGTGAGG | 320 | (102) |
|  |  | ial lower | GGAGGCCAACAATTATTTCC |  |  |
|  | *ipaH* | Shig-1 | TGGAAAAACTCAGTGCCTCT | 424 | (103) |
|  |  | Shig-2 | CCAGTCCGTAAATTCATTCT |  |  |
|  | *stx1* | stx1F | CACAATCAGGCGTCGCCAGCGCACTTGCT | 606 | (48) |
|  |  | stx1R | TGTTGCAGGGATCAGTGGTACGGGGATGC |  |  |
|  | *stx2* | stx2F | CCACATCGGTGTCTGTTATTAACCACACC | 372 | (47) |
|  |  | stx2R | GCAGAACTGCTCTGGATGCATCTCTGGTC |  |  |
| **ExPEC primers** | *focG* | focG_106F | CGTACCTGTACCATTGGTAATGGAGG | 366 | (51) |
|  |  | focG_471R | TGAATTAATACTTCCCGCACCAGC |  |  |
|  | *kpsMII* | kpsMII_121F | GCGCATTTGCTGATACTGTTG | 452 |  |
|  |  | kpsMII_572 | GGGAACATGATGCAGGAGATG |  |  |
|  | *papA* | papA_67F | ATGGCAGTGGTGTCTTTTGGTG | 717 |  |
|  |  | papA_+202R | CGTCCCACCATACGTGCTCTTC |  |  |
|  | *sfaS* | sfaS_210F | GTCTCTCACCGGATGCCAGAATAT | 138 |  |
|  |  | sfaS_347R | GCATTACTTCCATCCCTGTCCTG |  |  |
|  | *afa* | afa F | GGCAGAGGGCCGGCAACAGGC | 594 |  |
|  |  | afa R | CCCGTAACGCGCCAGCATCTC |  |  |
|  | *hlyD* | hlyD_92F | CTCCGGTACGTGAAAAGGAC | 904 |  |
|  |  | hlyD_995R | GCCCTGATTACTGAAGCCTG |  |  |
|  | *iutA* | iutA_674F | ATCGGCTGGACATCATGGGAAC | 314 |  |
|  |  | iutA_987R | CGCATTTACCGTCGGGAACGG |  |  |
