# Supplementary material for: ESBL Producing Escherichia coli in Faecal Sludge Treatment Plants: An Invisible Threat to Public Health in Rohingya Camps, Cox's Bazar, Bangladesh
Source: Front Public Health. 2021 Dec 15;9:783019. doi: 10.3389/fpubh.2021.783019 (PMC8714839; doi:10.3389/fpubh.2021.783019)
Supplement: Supplementary file 2 [file Table_2.DOCX]

**Supplementary Table 2.** Colony forming units of *E. coli* from the FSM plants of different technologies. Technologies are: UPF= Upflow Filter, CWL= Constructed Wetland, ABR= Anaerobic Baffled Reactor, LSP= Lime Stabilization Pond, WSP= Wastewater Stabilization Pond, DEWATS= Decentralized Wastewater Treatment Systems

| **Serial No.** | **1^st^ round** | | | | **2^nd^ round** | | | | **3^rd^ round** | | | |
| --- | --- | --- | --- | --- | --- | --- | --- | --- | --- | --- | --- | --- |
|  | Type of technology | Plant lab ID | Unit | *E. coli*  (cfu/100ml) | Type of technology | Plant lab ID | Unit | *E. coli*  (cfu/100ml) | Type of technology | Plant lab ID | Unit | *E. coli*  (cfu/100ml) |
| 1 | UPF | FSM-01 | Inlet | 7.40E+05 | ABR | FSM-05 | Inlet | 3.00E+07 | ABR | FSM-05 | Inlet | 1.50E+06 |
|  |  | FSM-02 | outlet | 3.00E+05 |  | FSM-06 | outlet | 1.00E+06 |  | FSM-06 | outlet | 0 |
| 2 | CWL | FSM-03 | Inlet | 3.20E+06 | ABR | FSM-11 | Inlet | 2.00E+08 | ABR | FSM-11 | Inlet | 1.60E+07 |
|  |  | FSM-04 | outlet | 1.72E+05 |  | FSM-12 | outlet | 1.00E+04 |  | FSM-12 | outlet | 2.90E+04 |
| 3 | ABR | FSM-05 | Inlet | 1.38E+08 | CWL | FSM-15 | Inlet | 4.00E+05 | CWL | FSM-15 | Inlet | 1.00E+04 |
|  |  | FSM-06 | outlet | 3.30E+04 |  | FSM-16 | outlet | 2.00E+06 |  | FSM-16 | outlet | 1.20E+03 |
| 4 | LSP | FSM-07 | Inlet | 2.00E+04 | UPF | FSM-17 | Inlet | 1.00E+06 | UPF | FSM-17 | Inlet | 3.50E+04 |
|  |  | FSM-08 | outlet | 1.90E+04 |  | FSM-18 | outlet | 0 |  | FSM-18 | outlet | 1.00E+03 |
| 5 | ABR | FSM-11 | Inlet | 2.00E+06 | LSP | FSM-21 | Inlet | 5.00E+06 | LSP | FSM-21 | Inlet | 5.50E+05 |
|  |  | FSM-12 | outlet | 6.60E+04 |  | FSM-22 | outlet | 3.70E+03 |  | FSM-22 | outlet | 6.00E+04 |
| 6 | ABR | FSM-13 | Inlet | 4.00E+06 | LSP | FSM-23 | Inlet | 2.10E+04 | LSP | FSM-23 | Inlet | 2.00E+06 |
|  |  | FSM-14 | outlet | 0 |  | FSM-24 | outlet | 3.40E+04 |  | FSM-24 | outlet | 2.00E+05 |
| 7 | CWL | FSM-15 | Inlet | 8.00E+05 | UPF | FSM-25 | Inlet | 1.38E+07 | UPF | FSM-25 | Inlet | 1.40E+05 |
|  |  | FSM-16 | outlet | 3.20E+05 |  | FSM-26 | outlet | 4.00E+03 |  | FSM-26 | outlet | 1.00E+04 |
| 8 | UPF | FSM-17 | Inlet | 1.00E+07 | ABR | FSM-29 | Inlet | 6.10E+05 | ABR | FSM-29 | Inlet | 1.40E+07 |
|  |  | FSM-18 | outlet | 0 |  | FSM-30 | outlet | 7.00E+03 |  | FSM-30 | outlet | 3.40E+03 |
| 9 | LSP | FSM-19 | Inlet | 1.00E+07 | UPF | FSM-33 | Inlet | 1.34E+06 | UPF | FSM-33 | Inlet | 3.40E+06 |
|  |  | FSM-20 | outlet | 8.00E+06 |  | FSM-34 | outlet | 4.40E+05 |  | FSM-34 | outlet | 3.00E+05 |
| 10 | LSP | FSM-21 | Inlet | 2.60E+07 | CWL | FSM-37 | Inlet | 9.30E+06 | CWL | FSM-37 | Inlet | 1.62E+07 |
|  |  | FSM-22 | outlet | 2.80E+04 |  | FSM-38 | outlet | 2.50E+07 |  | FSM-38 | outlet | 1.42E+07 |
| 11 | LSP | FSM-23 | Inlet | 4.40E+05 | WSP | FSM-39 | Inlet | 4.00E+06 | WSP | FSM-39 | Inlet | 2.00E+05 |
|  |  | FSM-24 | outlet | 0 |  | FSM-40 | outlet | 5.00E+02 |  | FSM-40 | outlet | 3.00E+02 |
| 12 | UPF | FSM-25 | Inlet | 7.20E+06 | DEWATS | FSM-41 | Inlet | 1.20E+05 | DEWATS | FSM-41 | Inlet | 1.10E+05 |
|  |  | FSM-26 | outlet | 2.10E+04 |  | FSM-42 | outlet | 4.90E+06 |  | FSM-42 | outlet | 4.00E+04 |
| 13 | LSP | FSM-27 | Inlet | 3.50E+06 | DEWATS | FSM-43 | Inlet | 4.10E+03 | DEWATS | FSM-43 | Inlet | 1.00E+07 |
|  |  | FSM-28 | outlet | 1.00E+07 |  | FSM-44 | outlet | 0 |  | FSM-44 | outlet | 0 |
| 14 | ABR | FSM-29 | Inlet | 2.00E+05 | DEWATS | FSM-45 | Inlet | 2.70E+07 | DEWATS | FSM-45 | Inlet | 3.00E+07 |
|  |  | FSM-30 | outlet | 1.00E+02 |  | FSM-46 | outlet | 4.00E+04 |  | FSM-46 | outlet | 2.00E+04 |
| 15 | ABR | FSM-31 | Inlet | 1.10E+07 | WSP | FSM-47 | Inlet | 3.00E+06 | WSP | FSM-47 | Inlet | 2.30E+06 |
|  |  | FSM-32 | outlet | 1.50E+05 |  | FSM-48 | outlet | 8.00E+04 |  | FSM-48 | outlet | 0 |
| 16 | UPF | FSM-33 | Inlet | 1.00E+07 | WSP | FSM-49 | Inlet | 2.30E+05 | WSP | FSM-49 | Inlet | 1.80E+07 |
|  |  | FSM-34 | outlet | 5.00E+05 |  | FSM-50 | outlet | 1.00E+04 |  | FSM-50 | outlet | 3.00E+05 |
| 17 | UPF | FSM-35 | Inlet | 6.30E+05 | CWL | FSM-51 | Inlet | 2.80E+05 | CWL | FSM-51 | Inlet | 7.00E+03 |
|  |  | FSM-36 | outlet | 3.10E+05 |  | FSM-52 | outlet | 1.55E+06 |  | FSM-52 | outlet | 5.00E+04 |
| 18 | CWL | FSM-37 | Inlet | 7.00E+06 | LSP | FSM-53 | Inlet | 2.00E+06 | LSP | FSM-53 | Inlet | 7.00E+05 |
|  |  | FSM-38 | outlet | 3.00E+04 |  | FSM-54 | outlet | 3.00E+03 |  | FSM-54 | outlet | 1.07E+06 |
